# A bio-fortified whole tomato food supplement as potential dietary tool for the management of Metabolic Dysfunction-Associated Steatotic Liver Disease (MASLD)

**DOI:** 10.1186/s12967-026-07907-7

**Published:** 2026-02-27

**Authors:** Pier Giorgio Natali, Luisa Imberti, Mauro Piantelli, Marco Minacori, Alessandra Sottini, Erica Gianazza, Cristina Banfi

**Affiliations:** 1 Mediterranean Task Force for Cancer Control (MTCC), via Pizzo Bernina, 14, 00141 Rome, Italy; 2 Section of Microbiology, University of Brescia, P. le Spedali Civili, 1, Brescia, Italy; 3 Department of Medicine and Aging Sciences, Center for Advanced Studies and Technology (CAST), G. D’Annunzio University, Via Luigi Polacchi, 11, 66100 Chieti, Italy; 4 Department of Bioscience and Agro-Food and Environmental Technology, University of Teramo, Campus “Aurelio Saliceti”, Via R. Balzarini, 1, 64100 Teramo, Italy; 5 Highly Specialized Laboratory, Service Department, ASST Spedali Civili of Brescia, P.le Spedali Civili, 1, 25123 Brescia, Italy; 6 Centro Cardiologico Monzino IRCCS, Unit of Functional Proteomics, Metabolomics, and Network Analysis, Via Privata Carlo Parea, 4, 20138 Milan, Italy

**Keywords:** Antioxidants, Functional foods, Lipids, Metabolic Dysfunction-Associated Steatotic Liver Disease, Tomato

## Abstract

**Background:**

Western diets, rich in refined fats and carbohydrates, are recognized as a major player in hepatic lipid accumulation in adults and youngsters, leading to the growing prevalence of Metabolic Dysfunction-Associated Steatotic Liver Disease (MASLD), formerly known as non-alcoholic fatty liver disease, the gate to cirrhosis and cancer. Due to the lack of approved therapies, antioxidant-rich dietary regimens targeting MASLD relevant pathologic pathways may be of more immediate translational impact. As tomatoes are a major globally accessible source of antioxidant/inflammatory nutrients, we have investigated whether a novel whole tomato-based food supplement (WTFS), possessing an effective antioxidant activity and hindering multiple metabolic pathways, can interfere with mechanisms fostering MASLD progression.

**Methods:**

Lipidomic and proteomic analyses were performed in the HepG2 liver human cell line treated with WTSF.

**Results:**

WTFS induces a marked reduction in triglycerides and cholesterol ester content, a decrease in the relative levels of diacylglycerols, lysophosphatidylcholine, lysophosphatidylethanolamines, phosphatidylethanolamines, and lower expression of transforming growth factor-α, tumor necrosis factor-like weak inducer of apoptosis (TWEAK), and Fms-related tyrosine kinase 3 ligand (FLT3LG), signaling relevant to MASLD progression.

**Conclusions:**

WTFS may represent a potential candidate for clinical trials in supplementing antioxidant-rich dietary regimens such as the healthy but hard-to-follow Mediterranean diet, the presently first-line preventive and therapeutic nutritional regimen for MASLD.

**Supplementary Information:**

The online version contains supplementary material available at 10.1186/s12967-026-07907-7.

## Background

Epidemiological, experimental, and clinical evidences increasingly support the protective role of antioxidants in liver diseases by regulating lipid homeostasis, lowering inflammation, and resulting scarring [1, 2]. Standing the dismetabolic origins of Metabolic Dysfunction-Associated Steatotic Liver Disease (MASLD) [3], functional foods [4], capable of modulating fat metabolism and associated inflammation, are currently undergoing scrutiny being environmentally sustainable and amenable to consumption by large populations fractions [5]. In this regard, increasing attention has been focused on the polyene lycopene, potent intrinsic [5] and inducing antioxidant [6] which decreases lipid peroxidation [7] and possesses anti-inflammatory [8] and lipid-lowering properties [9, 10]. Of relevance, fats lowering activity of this carotenoid relies on the dual mechanism of HMG-CoA reductase inhibition and downregulation of PCSK-9 mRNA synthesis [11]. Because of its hepatic accumulation [12], where active metabolites, i.e. apo-lycopenals and apo-lycopenones are generated [13], the lycopene-containing foods are attractive exploratory candidates to control the development and progression of MASLD, a disease of increasing incidence and prevalence [14]. To acquire the broad spectrum of its healthy biological properties, the trans isomeric form of the naturally occurring lycopene needs to be modified metabolically [15] in the cis configuration the only biologically active isomer [6] and by the concomitant uptake of other tomato micronutrients, such carotenoid [16], and nutrients generated by the cooking of the berry [17]. Indeed, the consumption of whole fruits has been shown to result in dose-dependant healthier effects than single lycopene supplementation in animal [18] and human studies [19]. Therefore, the unique combination of antioxidant and anti-inflammatory nutrients [20] with converging biological activities of the berry [21], which is the primary dietary source of antioxidants [22], advocates the choice of whole cooked tomato consumption as a functional food for equitable and sustainable healthy diets [21]. Along this line of investigation, a novel whole tomato (98%) food supplement (WTFS) [23], enriched (2%) with olive waste water in the form of an additives/ excipients-free powder [24], has been recently described which is characterized by a multi-nutrients composition [25] capable of interfering with metabolic pathways sustaining oxidative stress, chronic inflammation and neoplastic transformation [26] and with the potential of modulating gut dysbiosis, a relevant contributor to fat induced hepatitis [27–29].

To further establish whether this improved well defined functional food may be a candidate for clinical studies aimed at mitigating lipids liver accumulation, we have performed a lipidomic and proteomic analysis on HepG2 human liver cells exposed to the WTFS.

## Methods

### Cell cultures

The HepG2 human hepatoblastoma cells, a reference target to study liver function including lipid metabolism and hepatotoxicity [30, 31] obtained from the American Type Culture Collection (ATCC, Manassas, VA, USA), were plated at 1 × 10^6^ in T-75 cell culture flasks in minimum Essential Media supplemented with 10% heat-inactivated fetal calf serum containing 2 mmol/L L-glutamine, 100 IU/ml penicillin, 100 mg/ml streptomycin, 2.2 mg/L sodium bicarbonate, and 1 mmol/L sodium pyruvate, under a humidified atmosphere of 95% air/5% CO_2_ at 37° C, as previously described [32]. WTSF preparation in dimethyl sulfoxide (DMSO) and cells treatment followed the conditions described by Rubini et al. [33], HepG2 cells cultured with only DMSO were used as control (CTRL). Cells were collected as a dry pellet following washing with phosphate-buffered saline and subsequent counting. A precipitation solvent composed of water-saturated butanol and 20 mM ammonium acetate in methanol (in a 1:1 volume ratio) was added to achieve a concentration of 3,500 cells/µL. The solution was then sonicated and centrifuged for 5 min at 4,500 rpm at room temperature. The supernatant was then frozen at -80° C for later lipidomic analysis.

### Lipidomic analysis

Lipidomic analysis was conducted using a 5500 QTRAP LC-MS/MS system (AB Sciex, Framingham, MA, USA) equipped with an electrospray ionization source and coupled with an ExionLC HPLC system (AB Sciex). Samples were injected onto an Xbridge BEH C18 precolumn (3.5 μm, Waters Corporation, Milford, MA, USA) and subsequently separated using the Xbridge C18 (3.5 μm, 2.1 × 100 mm, Waters Corporation) analytical column. The injection volume was 1 µL for positive ion mode and 5 µL for negative ion mode. The column temperature was set at 50 °C, and elution was conducted at a flow rate of 0.400 mL/min by incrementally increasing the concentration of organic solvent B from 0% to 97% over 50 min. The solvent A was composed of 10 mM ammonium formate in a mixture of water, acetonitrile, and 2-propanol (50:30:20 v/v/v), while solvent B contained 10 mM ammonium formate in a mixture of water, acetonitrile, and 2-propanol (1:9:90 v/v/v). All samples were analyzed in triplicate in both positive and negative modes.

The multiple reaction monitoring analysis involved the detection of 333 transitions in positive ion mode, encompassing various lipid classes, including carnitines, cholesterol esters (CE), ceramides (Cer-d), cholesterol, diacylglycerols (DG), glucosylceramides (GCer), lactosylceramides (LacCer), lysophosphatidylserines (LPS), lysophosphatidylcholine (LysoPC), lysophosphatidylethanolamines (LysoPE), phosphatidylcholines (PC), phosphatidylethanolamines (PE), phosphatidylserines (PS), sphingomyelins (SM), and triacylglycerols (TG). In negative ion mode, 93 transitions were monitored, which included bile acids (BA), fatty acids, cardiolipins (CL), lysophosphatidic acids (LPA), lysophosphatidylinositol acids (LPI), phosphatidic acids (PA), phosphatidylinositol acids (PI), phosphatidylglycerol acids (PG), and sulfatides (Sul-d).

Data processing was conducted using MultiQuant software version 3.0.2 (AB Sciex), and statistical analysis was performed utilizing the R software package (version 4.3.2).

### Olink analysis

The analysis was conducted by using the Proximity Extension Assay Olink Target 48 Cytokine panel (Olink Proteomics, Uppsala, Sweden), providing absolute (pg/ml) measurements for the selected cytokines. A comprehensive list of the 45 analyzed proteins, along with their respective acronyms and UniProt codes, is provided in Supplemental Table S1. This approach uses specific antibody probes marked with dual oligonucleotides that bind to target proteins. Quantitative DNA detection follows, where the oligonucleotide sequence is amplified, via microfluidic real-time PCR. Quality control procedures and normalization were performed on cycle threshold data from both internal and external controls.

### Gene ontology analysis

Protein-protein interaction analysis and functional enrichment for Gene Ontology (GO) categories were performed using the STRING database v12.0 (https://string-db.org/). A set of input proteins was analyzed with the interaction confidence score set to default (medium confidence ≥ 0.4), and the maximum number of interactors was limited to no more than 5 to ensure a functionally relevant and interpretable network. Functional enrichment was evaluated under the GO Molecular Function category. The significance of enrichment was assessed using STRING’s built-in statistical framework, based on a modified Fisher’s exact test corrected for multiple testing (false discovery rate).

## Results

**Fig. 1:**
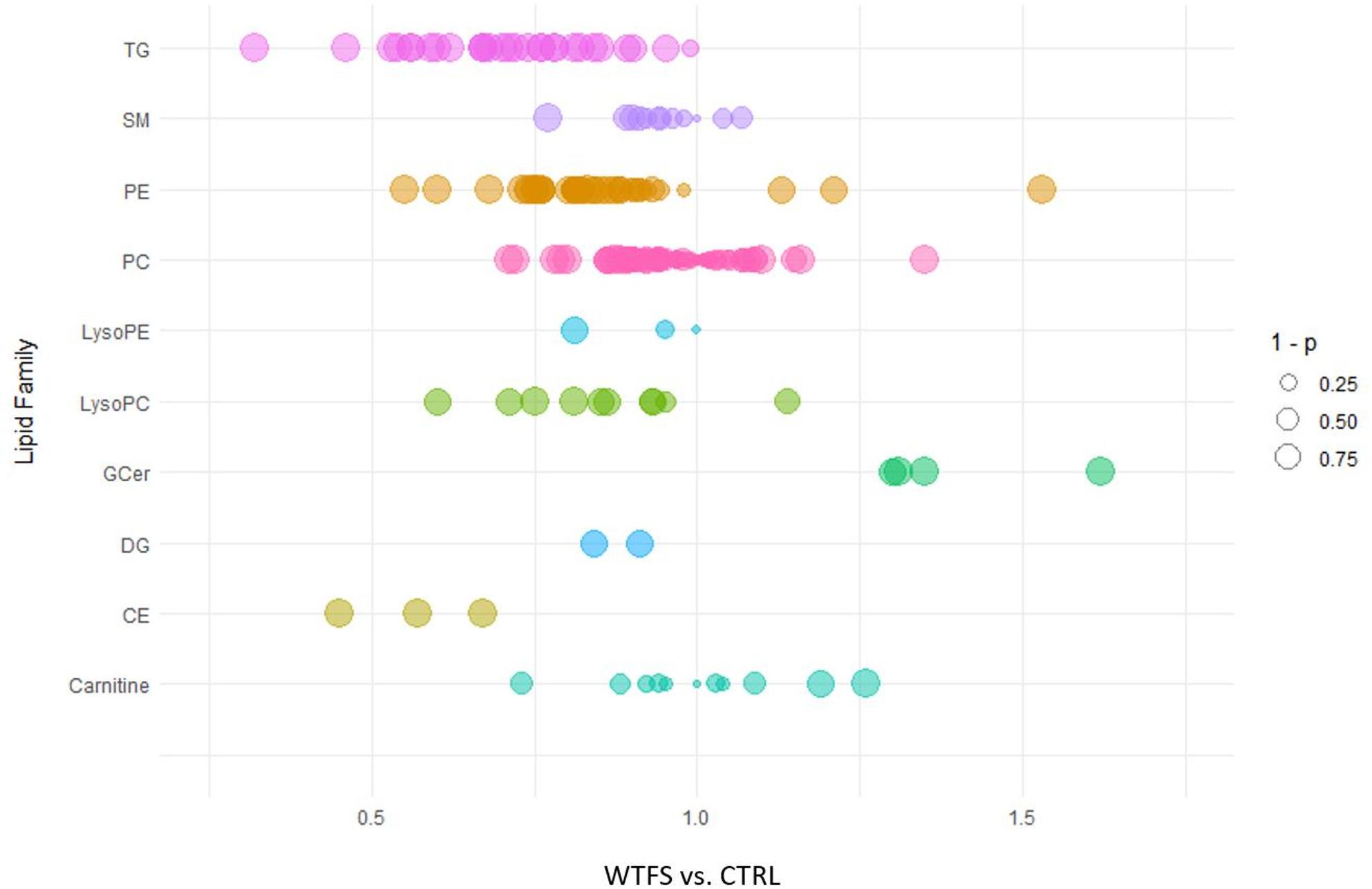
The bubble plot reports various lipid classes examined through targeted lipidomic analysis in positive ion mode. A notable reduction in the relative levels of cholesterol esters (CE), diacylglycerols (DG), lysophosphatidylcholine (LysoPC), lysophosphatidylethanolamine (LysoPE), and triacylglycerols (TG) was observed in cells treated with WTFS. A significant decrease was recorded in the levels of all TG and CE in the treated cells. The size of the bubbles corresponds to the significance of the fold changes (WTFS vs. CTRL), reported as 1 minus the p-values. The other lipid classes analyzed include glucosylceramides (GCer), phosphatidylcholines (PC), phosphatidylethanolamine (PE), and sphingomyelins (SM)

Targeted lipidomic analysis in positive ion mode was performed to assess the impact of WTFS treatment on the lipid composition of HepG2 cells. Figure 1 displays a bubble plot summarizing fold changes in lipid species abundance. Each bubble represents an individual lipid species, colored by lipid class. The x-axis denotes the fold change, while bubble size is proportional to statistical significance, represented as 1 minus the p-value. This visualization allows simultaneous assessment of both the magnitude and significance of lipid alterations across different classes.

As illustrated in Fig. 1, WTFS exposure resulted in a significant reduction in the relative abundance of multiple lipid classes compared to CTRL. Specifically, the levels of CE, DG, LysoPC, LysoPE, PE, and TG were decreased. Among these, TG and CE exhibited the most pronounced reduction, with an average fold change of 0.71 and 0.56, respectively. Conversely, a significant increase was observed in the levels of GCer, which displayed a mean fold change of 1.40. No appreciable alterations were detected in the abundance of carnitine, PC, or SM, indicating a selective remodeling of the lipid profile upon WTFS treatment.

**Fig. 2:**
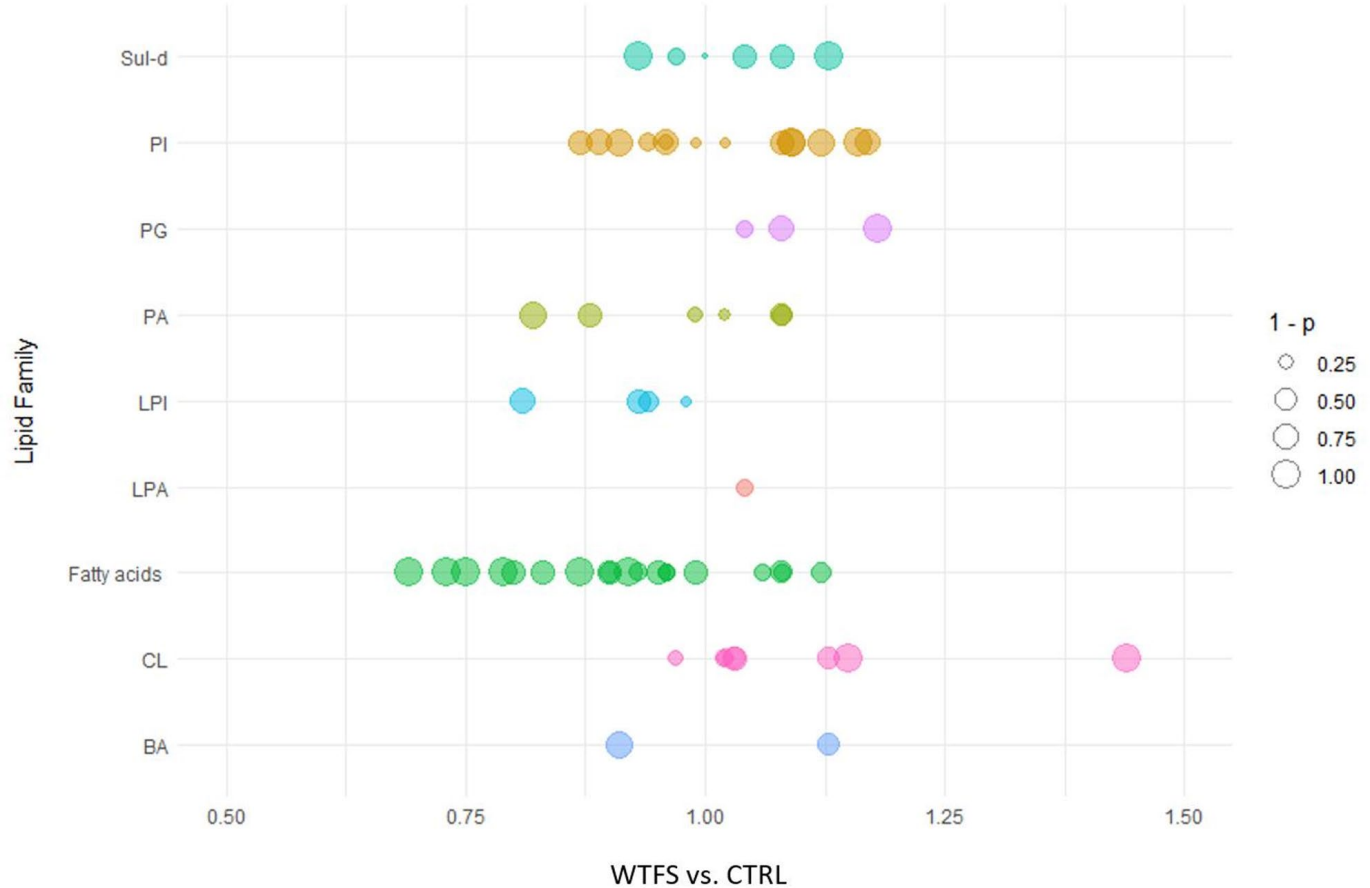
The bubble plot represents different lipid classes measured by targeted lipidomic analysis in negative ion mode. The size of the bubbles corresponds to the significance of the fold changes (WTFS vs. CTRL), reported as 1 minus the p-values. The lipid classes analyzed include bile acids (BA), cardiolipins (CL), lysophosphatidic acids (LPA), lysophosphatidylinositol acids (LPI), phosphatidic acids (PA), phosphatidylinositol acids (PI), phosphatidylglycerol acids (PG), and sulfatides (Sul-d)

In negative ion mode, treated cells with WTFS showed a decrease in the levels of fatty acids and LPI compared to CTRL (Fig. 2). Conversely, only CL and PG exhibited slight increases in the WTFS-treated cells. There were no measurable changes in the relative abundances of BA, PA, LPA, PI, and Sul-d.

Since cytokines and growth factors, known mediators of liver function, can contribute to the onset and progression of various liver diseases [34], we investigated the cytokines’ modulatory activity on HepG2 cells treated with WTFS.

**Fig. 3:**
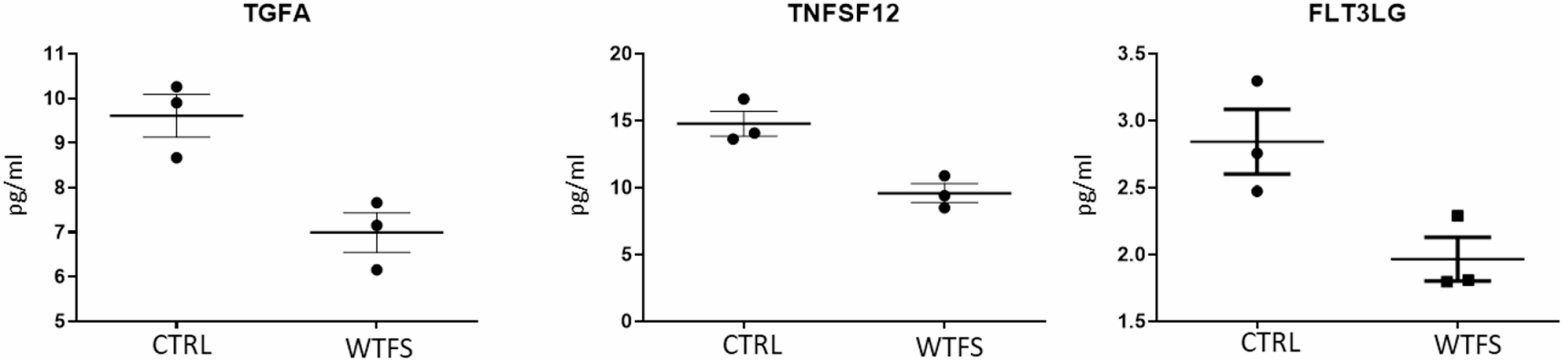
Targeted proteomic analysis was conducted on HepG2 cells treated with WTFS, utilizing the Olink Target 48 cytokine assay

According to the proteomic analysis (Fig. 3), three molecules out of the 45 proteins analyzed (see Table S1) exhibited significant downregulation by the WTFS: (a) TGFA, the epidermal growth factor (EGF) family member known as transforming growth factor-α (TGF-α), which plays a crucial role in modulating cell growth, differentiation, migration, and survival [35]; and (b) TNFSF12, also referred to as TNF-related weak inducer of apoptosis (TWEAK), a multifunctional cytokine with a diverse array of biological activities [36], which also serves as a ligand for the fibroblast growth factor-inducible 14 (Fn14) receptor; (c) FLT3LG, or Fms-related tyrosine kinase 3 ligand, that by binding the Flt3/CD135 receptor, induces dimerization and autophosphorylation of the receptor, and activates multiple downstream signaling pathways, including phosphatidylinositol-3 kinase (PI3K)/Akt/ mammalian target of rapamycin (mTOR), JAK/STAT pathways, and RAS/RAF/ extracellular signal-regulated kinase (ERK). These pathways are involved in the survival and proliferation of various cell lineages, including hepatocytes [34].

**Fig. 4:**
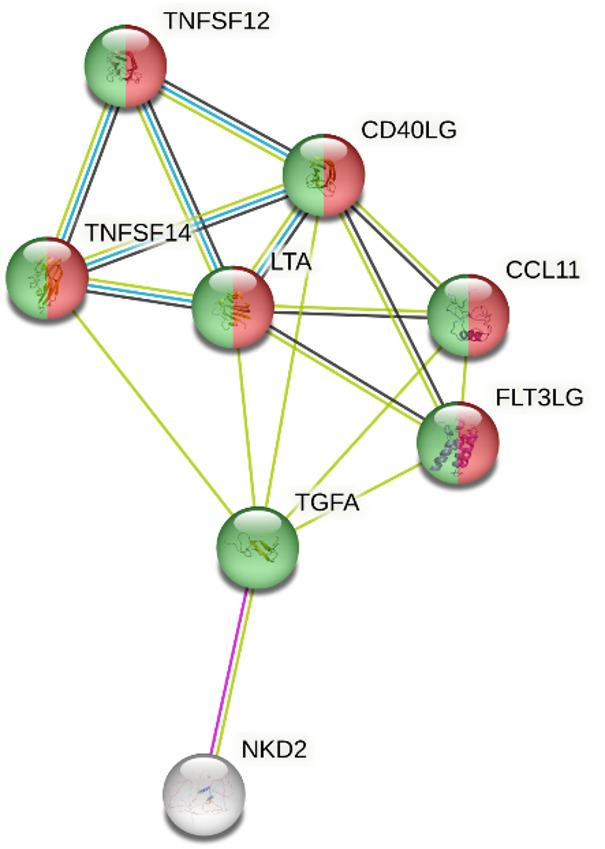
Protein-protein interaction network generated using STRING v12.0. Nodes represent proteins, while edges indicate predicted functional associations. Enriched GO Molecular Function terms are annotated with color highlights: red indicates “cytokine activity” and green indicates “receptor ligand activity”. The analysis was performed with a confidence threshold of 0.4 and a maximum of 5 interactors to maintain interpretability. Only significantly enriched categories are shown

The STRING network analysis, which identified functionally enriched molecular functions among the input proteins (max 5 interactors) showed two enriched GO Molecular Function terms: “cytokine activity” (GO: 0005125) indicating significant involvement of several nodes in cytokine-mediated signaling and “receptor ligand activity “(GO: 0048018), representing proteins with potential to act as ligands for receptor-mediated processes (Fig. 4).

## Discussion

MASLD is becoming globally a highly prevalent disease in the general population [37], especially in those patients affected by type 2 diabetes mellitus and obesity [38]. Of relevance, MASLD is also the most frequent pediatric liver disease [39, 40]. Although approved therapeutic regimens are currently unavailable [41], MASLD may be amenable to preventive and therapeutic interventions through the adherence to international guidelines recommending a lifestyle-based approach, relying on healthy diets [42]. This strategy can by variable extent modulate hepatic steatosis and counteract liver damage, preventing and delaying the evolution of MASLD to cirrhosis and cancer [43]. In this context, phytochemical and natural compounds are undergoing an in-depth investigation [44], among which carotenoids are of major interests [45]. Multidisciplinary evidences strongly indicate that lycopene [8], a potent and largely available dietary antioxidant/anti-inflammatory nutrient, may be beneficial in the management of MASLD as it has been proven to be efficacious in the case of non-alcoholic fatty liver disease [45].

On the other hand, evidence correlating either the single carotene or lycopene-containing foods, has so far failed to provide conclusive evidence of its protective efficacy on MASLD [46]. This limitation is likely to be multifactorial, as assignable to the wide range of individual variability in the metabolism of lycopene into its bioavailable cis configuration [15] and the need to resort to high consumption of lycopene-containing foods lacking defined nutrients profiles [47], often associated with high calories uptake. It should be underlined that lycopene with a daily requirement of 0.5 mg/kg and a plasma elimination half-life of 5 days [48, 49], taken in the range of 5 to 7 mg/day [50] is mainly deriving from lycopene/ E 160d red food coloring agent intake [49]. To overcome these limitations, an improved powder formulation [24] of this functional food has been developed using whole tomato fruits, not completely freed from peels and seeds [51]. This WTFS, produced by calibrated heating of the berry and spray drying [26] is biofortified with olive wastewater antioxidants and by the presence of Fru-His Amadori’s chelators [52] displaying an overall superior composition compared to available tomato commodities. WTFS has been shown to interfere with metabolic pathways mediating oxidative stress and inflammation, as demonstrated by in vitro [33], animal models [53], and human conditions of known susceptibility to tomato micronutrients benefits [25].

The information gathered in this study utilizing the hepatoblastoma cell line Hep2G [54] a widely used cell target for investigating hepatocyte-intrinsic metabolic processes [31], may be informative to explore strategies to contrast liver lipid storage but should be interpreted cautiously. Their transformed phenotype necessarily constrains extrapolation to normal hepatocyte biology. Accordingly, the findings reported here should be viewed as hypothesis-generating and require further analysis in more comprehensive physiologically relevant models. Indeed, although the progression of MASLD is the result of a stepwise engagement of other parenchymal, i.e. Kuppfer cells [55], stellate cells [56], and non-parenchymal, i.e. immune cells [57], the driver of this progressive disease stems from the triglycerides accumulation [58] and lipogenesis [59] in hepatocytes overrunning their dismissal capacity through fatty acid oxidation and/or higher production rates of very-low-density lipoprotein particles [60].

While targeted lipidomic profiling provides high-resolution quantitative information on intracellular lipid species, it does not directly assess lipid droplet abundance or morphology. Therefore, the lack represents a limitation of the present study. Nevertheless, triglycerides and cholesterol esters constitute the core components of hepatic lipid droplets, and changes in their intracellular levels are widely accepted as robust biochemical surrogates of lipid storage in hepatocyte-based models. In this context, the marked reduction of triglycerides and cholesterol esters observed following WTFS treatment is consistent with a decrease in intracellular lipid accumulation, although direct visualization was not performed. Future studies integrating lipidomic profiling with lipid droplet imaging and quantitative morphometric analyses will corroborate these findings and to more precisely define the impact of WTFS on hepatocellular lipid storage dynamics.

Although the ability of WTFS single components to downmodulate the lipidomic asset of HepG2 cells cannot be fully appreciated, a converging ability of lycopene and other WTFS components i.e. tocopherol, tyrosol, hydroxytyrosol, oleopeurina, the inhibition of STAT-3 [61], and AhR receptors activation [62], can be hypothesized [33].

Considering that WTFS has been shown to interfere with a several cell signaling, i.e. RTK receptor activation, nuclear factor-kappa B (NF-κB), mitogen-activated protein kinases (MAPK), upregulating the recruiting of inflammatory cells and inhibiting JAK/STAT kinases modulating inflammatory genes [33], the proteomic analysis also identified three major targets of WTFS in HepG2 cells, which, by variably activating different signaling, may foster the progression of MASLD. Indeed, a) TGF-α, by engaging the EGF receptor, triggers multiple downstream signaling, i.e. RTK, PI3K, ERK, and mTOR, which are relevant to liver regeneration [63] thus being a target of WTFS complex of micronutrients. In addition, TGF-α has been identified as an independent indicator of substantial liver fibrosis [64]. TWEAK, a mitogen for liver progenitor cells [65], while undetectable in normal liver, it is significantly upregulated in patients with fatty liver, thus offering a potential therapeutic target [66], also in view that lowering its in vivo signaling may decrease levels of inflammation [36] and unbalanced signaling may lead to altered tissue architecture [67]. Modelling of ligand and receptor interactions at multi cellular level [68] and integrative single-cell and spatial transcriptomic analyses have identified TNFRSF12A [69] as a relevant actor in supporting fibrinogenesis in liver pathology, thus a potential therapeutic target [70].

Activation of FLT3LG, which through binding to the receptor Flt3/CD135, causes dimerization and autophosphorylation of the receptor with activation of iPI3K/Akt/mTOR, JAK/STAT, and RAS/RAF/ERK pathways [71], concurs to fibrosis through epithelial mesenchymal transition [72].

In this regard, WTFS contains a complex of anti fibrogenic nutrients (lycopene, quercetin, narigenin, verbascoside) which can modulate epithelial-mesenchymal transition [73] and reduce platelets aggregation [74], more recently recognized as a relevant co-factor in liver fibrosis [75].

Collectively the proteomic modulation of TGF-α, TWEAK, and FLT3LG observed in WTFS-treated HepG2 cells should be interpreted with caution. While these mediators are well documented as contributors to inflammatory, regenerative, and fibrogenic signaling in MASLD and other chronic liver diseases, the present findings do not establish a direct causal relationship with disease progression. Rather, the downregulation of these factors identifies signaling nodes that are responsive to WTFS in a hepatocyte-centered model and that are biologically relevant in the broader context of MASLD pathophysiology. These results therefore provide associative evidence and generate mechanistically plausible hypotheses that warrant further validation in physiologically complex systems, including co-culture models, organoids, and in vivo studies.

Gene Ontology analyses revealed significant functional enrichment among the input proteins, particularly in key molecular functions such as “cytokine activity” and “receptor ligand activity”. The enrichment of cytokine activity, supported by the clustering of multiple nodes, suggests a prominent involvement of cytokine-mediated signaling pathways in the biological context under investigation. Collectively, these enriched terms point to a functional network characterized by intercellular communication and signal transduction. However, it should be noted that the STRING network and Gene Ontology analyses were performed on a limited set of differentially expressed proteins and should therefore be interpreted with caution. While this constrained dataset limits the robustness of pathway-level inferences, the analysis was intended to provide functional context rather than a comprehensive reconstruction of signaling pathways.

Accordingly, the identification of enriched molecular functions, such as cytokine activity and receptor ligand activity, reflects coherent functional convergence among the WTFS-modulated proteins and supports their relevance in intercellular signaling processes implicated in MASLD. These findings are best regarded as qualitative and hypothesis-generating, highlighting biologically meaningful associations that warrant validation in broader proteomic or systems-level studies.

The conclusions of the present work are intentionally framed to reflect a hepatocyte-centered, hypothesis-generating study. While the data do not allow definitive mechanistic or translational claims regarding MASLD progression or fibrosis, they identify WTFS as a nutritionally relevant candidate capable of modulating lipid and cytokine-related pathways that are central to the natural history of MASLD. A constant dietary supplementation with WTFS containing a complex of highly bioactive nutrients which share biological activities with lycopene may have, on the other hand, in vivo healthy effects that go beyond those produced on hepatocytes since it can modulate high-density lipoprotein [76], and potentially capable of interfering with multiple signaling relevant to progression of MASLD because upregulated in variety of cell types contributing to inflammation, angiogenesis, and fibrosis.

It should be noted that WTFS has been shown to modulate signaling of a wide array of cytokines and chemokines in animal models [53], and to inhibit STAT-3 activation involved in non-alcoholic fatty liver disease progression [77]. Furthermore, the WTFS content of highly bioavailable cis lycopene may be advantageous in patients with MASLD whose liver has impaired ability to generate lycopene active metabolites (i.e. apo-lycopenals and apo-lycopenones [13]. It should be also underlined that of lycopene and tomato-based supplements are emerging of potential clinical usefulness in the management of fatty liver associated disease in animals [78] and humans [79–81]. Since available murine models mimicking MASLD do not mirror closely the human condition [82], WTFS at its stage of development, can be regarded as an advanced nutritional candidate for human interventional studies to critically establish the potential costs/benefits [25] of this biofortified side-effects free functional food in the management of MASLD.

These studies can be aimed at improving available therapeutic regimens of not yet optimal performance [83] or still ongoing long-term assessment [84]. This also in view that a prototype of WTFS has been shown clinical benefit as Food for Special Medical Purposes [85].

From the immediate translational point of view [86], WTFS appears an “ad hoc” functional food supplement to complementing the Mediterranean diet, highly recommended for the prevention/treatment of non-alcoholic fatty liver disease [87–89], but now recognized as hard to follow [90], thus hampering its wide compliance and deriving benefits by large population fractions [91]. These will include individuals with glucose intolerance [92], in whom MASLD is often co-existing [93] but refrain from consuming high-calorie tomato-seasoned dishes, the main source of adequate amounts of bioavailable antioxidants carotenoids whose deficiency has been described in fatty liver [94].

## Supplementary Information

Below is the link to the electronic supplementary material.

Supplementary Material 1

## Data Availability

The datasets analyzed during the current study are available from the corresponding author upon reasonable request.

## Abbreviations

CTRL: Control
DMSO: Dimethyl sulfoxide
MASLD: Metabolic Dysfunction-Associated Steatotic Liver Disease
WTFS: Whole tomato-based food supplement
